# POStoperative INTELLiVENT-adaptive support VEntilation in cardiac surgery patients (POSITiVE) II—study protocol of a randomized clinical trial

**DOI:** 10.1186/s13063-024-08296-2

**Published:** 2024-07-03

**Authors:** Martin H. Bernardi, Dominique Bettex, Laura A. Buiteman–Kruizinga, Ashley de Bie, Matthias Hoffmann, Janine de Kleijn, Simon Corrado Serafini, Manon A. Molenaar, Frederique Paulus, Jasminka Peršec, Ary Serpa Neto, Reto Schuepbach, Paolo Severgnini, Andrej Šribar, Marcus J. Schultz, Edda Tschernko, for the POSITiVE II–investigators

**Affiliations:** 1 Department of Anesthesia, General Intensive Care and Pain Management––Division of Cardiothoracic and Vascular Anesthesia & Critical Care Medicine, Medical University of Vienna, Währinger Gürtel 18–20, 1090 Vienna, Austria; 2 University Hospital Zurich and University of Zurich, Zurich, Switzerland; 3 Department of Intensive Care, Amsterdam UMC, Location AMC, Amsterdam, the Netherlands; 4 Department of Intensive Care, Reinier de Graaf Hospital, Delft, the Netherlands; 5 Department of Intensive Care, Catharina Hospital Eindhoven, Eindhoven, the Netherlands; 6 Department of Surgical Sciences and Integrated Diagnostics (DISC), University of Genoa, Genoa, Italy; 7 Clinical Department of Anesthesiology, Resuscitation and Intensive Care Medicine, University Hospital Dubrava, Zagreb, Croatia; 8 Department of Critical Care Medicine, Hospital Israelita Albert Einstein, Sao Paolo, Brazil; 9 Australian and New Zealand Intensive Care Research Centre (ANZIC-RC), School of Public Health and Preventive Medicine, Monash University, Melbourne, Australia; 10 Department of Intensive Care, Austin Hospital, Melbourne Medical School, University of Melbourne, Melbourne, Australia; 11 Cardiac Surgery Intensive Care Unit, ASST Dei Sette Laghi, University of Insubria, Varese, Italy

**Keywords:** Intensive care, Mechanical ventilation, Invasive ventilation, Postoperative ventilation, Cardiac surgery, Randomized clinical trial, Automation, Closed-loop, INTELLiVENT-ASV, I-ASV

## Abstract

**Background:**

One single-center randomized clinical trial showed that INTELLiVENT-adaptive support ventilation (ASV) is superior to conventional ventilation with respect to the quality of ventilation in post-cardiac surgery patients. Other studies showed that this automated ventilation mode reduces the number of manual interventions at the ventilator in various types of critically ill patients. In this multicenter study in patients post-cardiac surgery, we test the hypothesis that INTELLiVENT-ASV is superior to conventional ventilation with respect to the quality of ventilation.

**Methods:**

“POStoperative INTELLiVENT-adaptive support VEntilation in cardiac surgery patients II (POSITiVE II)” is an international, multicenter, two-group randomized clinical superiority trial. In total, 328 cardiac surgery patients will be randomized. Investigators screen patients aged > 18 years of age, scheduled for elective cardiac surgery, and expected to receive postoperative ventilation in the ICU for longer than 2 h. Patients either receive automated ventilation by means of INTELLiVENT-ASV or ventilation that is not automated by means of a conventional ventilation mode. The primary endpoint is quality of ventilation, defined as the proportion of postoperative ventilation time characterized by exposure to predefined optimal, acceptable, and critical (injurious) ventilatory parameters in the first two postoperative hours. One major secondary endpoint is ICU team staff workload, captured by the ventilator software collecting manual settings on alarms. Patient-centered endpoints include duration of postoperative ventilation and length of stay in ICU.

**Discussion:**

POSITiVE II is the first international, multicenter, randomized clinical trial designed to confirm that POStoperative INTELLiVENT-ASV is superior to non-automated conventional ventilation and secondary to determine if this closed-loop ventilation mode reduces ICU team staff workload. The results of POSITiVE II will support intensive care teams in their choices regarding the use of automated ventilation in postoperative care of uncomplicated cardiac surgery patients.

**Trial registration:**

Clinicaltrials.gov NCT06178510. Registered on December 4, 2023.

**Supplementary Information:**

The online version contains supplementary material available at 10.1186/s13063-024-08296-2.

## Background

Postoperative ventilation, often needed following cardiac surgery, may injure the lungs [1]. Lung-protective ventilation consists of ventilation using a properly-sized tidal volume (*V*_T_) to prevent volutrauma and barotrauma [2, 3], low pressures and energy to avoid energy trauma [4–7], and low levels of oxygen in inspired air (FiO_2_) to minimize the risk of chemotrauma [8, 9]. Applying lung-protective ventilation is challenging and time-consuming because it requires complex titrations of ventilator settings according to a patient’s continuously changing individual needs [10], which is particularly true for post-cardiac surgery patients scheduled for fast-track weaning from mechanical ventilation.

Automated ventilation is increasingly attractive for clinical use [11] as it improves the quality of ventilation and potentially offers support to diminished ICU team staff [12–14]. With automated ventilation, ventilator settings that are typically under control of the ICU team staff are under control of algorithms that form the basis of the automated ventilation mode. INTELLiVENT-ASV, one sophisticated automated ventilation mode, targets ventilation and oxygenation goals based on lung mechanics, whereby it automatically adjusts, breath-by-breath, *V*_T_, respiratory rate (RR), positive end-expiratory pressure (PEEP), and FiO_2_ [15]. Several studies have shown INTELLiVENT-ASV to be safe and effective with regard to lung-protective ventilation [16] and one single-center randomized clinical trial named “POStoperative INTELLiVENT-adaptive support VEntilation in cardiac surgery patients” (POSITiVE), showed INTELLiVENT-ASV to be superior to non-automated ventilation with respect to quality of ventilation in patients receiving postoperative ventilation after cardiac surgery [17].

Several studies showed that INTELLiVENT-ASV reduces the number of manual interventions by intensive care unit (ICU) team staff in various types of patients [12–14]. It cannot yet be concluded though if INTELLiVENT-ASV reduces ICU team staff workload since workload may also involve other aspects of care, such as but not limited to presetting the ventilator for use of automated ventilation, the number of blood gas analysis needed, the time spend with monitoring ventilatory support, and the number of and responses to alarms.

The here proposed randomized clinical trial, the successor of the abovementioned single-center POSITiVE study, will test the hypotheses that automated ventilation by means of INTELLiVENT-ASV is superior to non-automated ventilation by means of conventional ventilation with respect to quality of ventilation in postoperative ventilation in patients after cardiac surgery. We further hypothesize that INTELLiVENT-ASV reduces the ICU team staff workload and is non-inferior to, i.e., as good as non-automated ventilation with respect to duration of ventilation and length of stay in ICU.

## Methods

### Objectives and design

The “POStoperative INTELLiVENT-adaptive support VEntilation in cardiac surgery patients II” (POSITiVE II) study is an investigator-initiated, international, multicenter, parallel, randomized clinical trial that compares automated ventilation by means of INTELLiVENT-ASV with ventilation that is not automated by means of conventional ventilation in patients planned for elective cardiac surgery and expected to need postoperative ventilation in an intensive care unit for at least 2 h.

The primary objective is to compare the two ventilation strategies with respect to quality of ventilation. One major secondary objective is to compare the two ventilation strategies with respect to ICU team staff workload, including manual ventilator settings and alarms; we will also compare the two ventilation strategies with respect to patient-centered endpoints like duration of ventilation, and length of stay in ICU.

The study will be conducted according to the Good Clinical Practice guidelines and comply with the principles of the Declaration of Helsinki, national and international regulatory requirements, and general data protection regulations. The study is registered in a public registry, and a finalized statistical analysis plan will be prepublished before cleaning and closing of the database.

### Study population

POSITiVE II will include patients planned for elective cardiac surgery and to receive postoperative ventilation in an ICU.

In order to be eligible to participate in this study, a patient must meet the following criteria: aged above 18 years of age, scheduled for elective cardiac surgery, and expected to receive postoperative ventilation in the ICU for longer than 2 h. Before surgery, a potential patient who meets any of the following criteria will be excluded from participation in this study if: (i.) the surgical procedure concerned an emergency or semi-elective intervention (precluding informed written consent) or (ii.) any surgery other than coronary artery bypass grafting (CABG), valve replacement or repair, or a combination of these (i.e., patients planned for surgery for congenital heart disease, or scheduled for heart transplantation, will be excluded). Patients are also excluded if: (iii.) enrolled in another interventional trail; (iv.) no written informed consent is obtained; (v.) in case of a history of recent pneumectomy or lobectomy; or (vi.) COPD with oxygen at home; (vii.) has a body mass index higher than 35; or (viii.) a preoperative forced expiratory volume in the first second (FeV1)/forced vital capacity (VC) of less than 50% (if available); or (ix.) a preoperative arterial oxygen partial pressure (PaO2) < 60 mm Hg (at room air); or (x.) preoperative arterial carbon dioxide partial pressure (PaCO2) of more than 50 mm Hg; or (xi.) preoperative left ventricular ejection fraction of less than 30% (if available); or (xii.) preoperative systolic pulmonary artery pressure higher than 60 mm Hg (if available); or (xiii.) preoperative left ventricular mechanical support, e.g., Impella®; or (xiv.) preoperative use of veno-venous or veno-arterial extracorporeal support (ECMO). At the end of surgery, patients are additionally excluded if: (xv.) weaning from intraoperative extracorporeal support is not possible due to surgical complications or (xvi.) a cardiac assist device is implanted.

### Standard ventilation management

In the ICU, subjects are cared for by dedicated board-certified ICU team. Changes in treatment are implemented based on observations by ICU team and physicians and according to the recommendations in the local guideline for postoperative care. Automated ventilation is generally initiated within 10 min after ICU arrival, with automated adjustments of minute volume, PEEP, and FiO_2_ activated. Extubation is performed according to criteria described in the local protocol.

After arrival in the ICU, the attending caregiver initiates the ventilator mode of choice, using lung-protective ventilator settings, guided by blood gas analyses. Use of another automated mode is *not* allowed: only standard pressure-controlled ventilation or volume-controlled ventilation modes are allowed in passive patients and assisted breathing in active patients. *V*_T_ and RR, and PEEP and FiO_2_ are set and adjusted to keep etCO_2_ and PaCO_2_ in range. Typically, PEEP is limited to 5 to 12 cm H_2_O, FiO_2_ is limited to 30 to 100%. In case of a difference between PaCO_2_ and etCO_2_ of ≥ 5 mm Hg, the etCO_2_ target is adjusted. As soon as a patient displays spontaneous breathing efforts, the ventilator is switched from controlled to supported ventilation. The patient is extubated according to the local protocol; quantitative neuromuscular monitoring (e.g., train-of-four (TOF) monitoring) should be considered in case multiple doses of muscle relaxants are given in the operating room, or in the ICU––in those cases, before extubation residual curarization should be addressed (e.g., TOF > 0.9).

### Intervention

The intervention tested is named “INTELLiVENT-ASV”, an automated ventilation mode available at Hamilton Medical AG ventilators (Hamilton Medical AG, Bonaduz, Switzerland) and intended to be used in patients that need ventilation in an ICU. INTELLiVENT-ASV is a fully automated mode of ventilation, targeting the lowest work and force of breathing, through breath-by-breath adaptation of *V*_T_ and RR, and PEEP and FiO_2_ based on patient activity, airway pressures and continuous pulse oximetry readings and end-tidal carbon dioxide monitoring. It also uses a weaning protocol and spontaneous breathing trials to facilitate weaning.

After arrival in the ICU, subjects start as soon as possible with INTELLiVENT-ASV. The attending caregiver initiates this automated ventilation mode, typically within 10 min after arrival in the ICU, when the results of the first blood gas analyses become available. Automatic adjustments for percentage of alveolar minute volume, PEEP, and FiO_2_ are activated and standard thresholds are set: PEEP is limited to 5 to 12 cm H_2_O, FiO_2_ is limited to 30 to 100%, and a “normal lung condition” is chosen. In case of a difference between PaCO_2_ and etCO_2_ of ≥ 5 mm Hg, the etCO_2_ target is shifted as described in the clinical guideline in use in the participating ICU. If clinically indicated, the target range for SpO_2_ can be manually adjusted. The options “Quick Wean” and “automated spontaneous breathing trials” are activated at start of ventilation by means of INTELLiVENT-ASV.

With INTELLiVENT-ASV, manually switching off one or more of the automated controllers by the attending caregiver (i.e., due to poor sensor signal quality) is allowed at any point during the study, in compliance with standard ventilation management of the participating ICU. According to a standard operating protocol, in case of a large discrepancy between SpO_2_ and SaO_2_ (SaO_2_ > 5% lower than SpO_2_), the automated FiO_2_ and PEEP controllers should be switched off, and in case of a minute ventilation of > 200% of the predicted minute volume, the minute volume controller should be switched off.

### Standard procedures beyond ventilator management

Routine general anesthesia is performed according to the specific expertise and routine clinical use of each participating center. General anesthesia achieved by using both volatile anesthetics and intravenous anesthetics are both allowed.

Postoperative pain management, sedation, physiotherapeutic procedures, and fluid management will be performed in the intra-operative and/or post-operative period according to the specific expertise and routine clinical use of each center. There are no restrictions regarding concomitant care during the trial. The investigators suggest to adhere to the enhanced recovery after surgery (ERAS) guidelines (http://erassociety.org/guidelines/list-of-guidelines/): (1) to perform postoperative pain management in order to achieve a VAS pain score below 4 and regional or neuraxial analgesia can be used whenever indicated; (2) to use physiotherapy by early mobilization, deep breathing exercises with and without incentive spirometry, and stimulation of cough in the postoperative period; (3) to avoid fluid overload (according to the discretion of the attending anesthesiologist in the operating room and in the ICU); and (4) to use appropriate prophylactic antibiotics according to institutional standards. Furthermore, regarding surgical perioperative procedures, the investigators suggest adhering to the Safe Surgery Checklist from the World Health Organization (Safe surgery [www.who.int]).

### Minimization of bias

In order to prevent dropouts, randomization is performed shortly before the start of surgery as soon as it is certain the surgery will proceed and there is the availability of a ventilator that can be used for the study. Randomization will then be performed by local investigators using the Castor platform [www.castoredc.com]. Included patients will be randomly allocated in a 1:1 ratio to the INTELLiVENT group or to non-automated ventilation group. The allocation sequence will be computer-generated, using the Castor randomization module with permuted blocks of different block sizes, with a maximum block size of 8 and stratified per center.

Further minimization of bias will be achieved by involving at least two independent investigators per site. Investigator responsible for the preoperative and postoperative care cannot be kept blind due to the nature of the intervention tested. All analyses are preplanned, i.e., before closing of the database, and will be performed by an investigator that remains blinded for treatment allocation.

### Study endpoints

The primary outcome is quality of ventilation, which is the proportion of *time* spent in three predefined and previously used zones of ventilation (Appendix 1) in the first 2 h of postoperative ventilation, as used before in previous studies of automated ventilation [17, 18].

Zones of ventilation used to define the primary outcome: for tidal volume (VT ml/kg PBW), critical denotes over 12, acceptable spans 8 to 12, and optimal falls at or below 8; regarding maximum inspiratory pressure (Pmax, cm H2O), critical signifies 36 or higher, acceptable encompasses 31 to 36, and optimal is 30 or less. End-tidal CO2 (etCO2, mmHg) falls into critical if below 25 or at 51 and above, acceptable between 25 and 30 or 46 and 51 and optimal between 30 and 46. Finally, oxygen saturation (SpO2, %) is critical under 85, acceptable if over 98 or between 85 and 93, and optimal between 93 and 98, or over 93 with FiO2 ≤ 40%.

As an add to the primary endpoint, next to *time* spent in predefined zones, we will also calculate *proportion* of breath in predefined zones as secondary endpoint. Also, we will measure ICU team staff workload, which is captured by the ventilator software collecting data on alarms (number of alarms, types of alarm, duration of alarm, responses to alarm, alarm settings and adjustments, breath-by-breath alarm data, and any manual intervention at the ventilator) during postoperative care in the ICU (Appendix 2), as used before in previous studies [12–14, 19].

Other secondary endpoints are duration of postoperative ventilation and ICU length of stay. In addition, we will collect and compare patient–ventilator asynchrony requiring deepening of sedation and/or administration of muscle relaxants; hospital length of stay; and mortality in ICU and hospital.

### Study visits and data collection

**Fig. 1:**
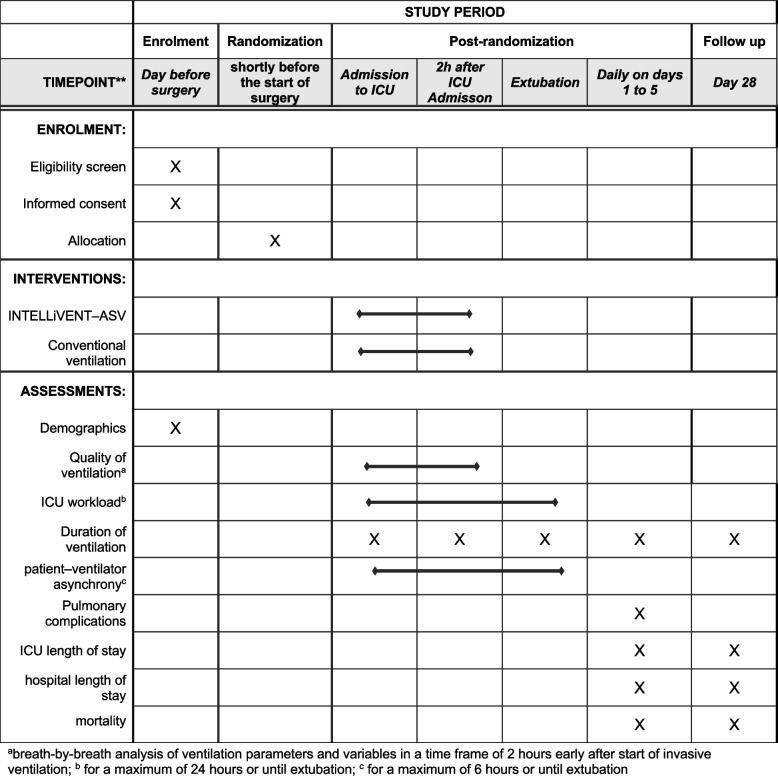
Schedule of enrolment, interventions, and assessments. **a** breath-by-breath analysis of ventilation parameters and variables in a time frame of 2 hours early after start of invasive ventilation; **b** for a maximum of 24 hours or until extubation; **c** for a maximum of 6 hours or until extubation

Patients scheduled for cardiac surgery in participating centers will be screened for eligibility by anesthesia staff or research personnel to whom this task has been delegated. Patient characteristics of screened patients meeting the inclusion criteria will be recorded, including age category of the subject, date of screening, and reason for not enrolling (Fig. 1).

Patients will be assessed before and during surgery, on postoperative days in the ICU, and the day before hospital discharge. Clinical data, ventilation data, and of outcomes will be captured within this timeframe.

Preoperative variables will be collected at pre-anesthetic visit or before induction of general anesthesia. The following variables are recorded—sex; age; height; weight; functional status; physical status (according to the American Society of Anesthesiologists score); frailty score (if available); cardiac status (heart failure, according to the New York Heart Association score, acute coronary syndrome, or persistent ventricular tachyarrhythmia’s); chronic obstructive pulmonary disease (if inhalation therapy and/or systemic steroids are used); respiratory infection in the last month; smoking status; type of cardiac surgery (CABG, valve reconstruction, valve replacement, combined CABG and valve surgery) and the surgical approach (sternotomy, hemi-sternotomy, thoracotomy, or minimal invasive procedure); and vital parameters (tympanic temperature, respiratory rate, oxygen saturation (SpO_2_), blood pressure, heart rate).

The patients will be assessed daily until the day after successful extubation, as well as on the last day before ICU and hospital discharge. In cases of re-intubation, timing of and reasons for re-intubation will be recorded. One investigator, blinded to the randomized intervention, will collect postoperative variables. The following variables are recorded on the first 5 days after surgery and the day before discharge: patient status (location, i.e., in hospital or at home—alive or deceased, and in the case of the latter the day of dying); occurrence of postoperative pulmonary complications; occurrence of extra pulmonary complications; requirement and duration of postoperative mechanical ventilation; re-admission to the intensive care unit (ICU) within the current hospitalization after initial ICU discharge; unplanned admission to another monitored ward; physiotherapy (e.g., for early mobilization, deep breathing exercise or stimulation of cough); urine output; need of renal replacement therapy; vital parameters including heart rate, tympanic temperature, oxygen saturation, respiratory rate, visual analogue scale pain score and blood pressure; impairment of wound healing and surgical wound infection; and blood tests including glucose, urea, creatinine, hemoglobin, C-reactive protein, and white blood cell count.

High-granular ventilation data will be captured using data storage devices or a dedicated laptop connected directly to the communication port at the ventilator:

- Breath-by-breath ventilation data is captured—data collection continues until tracheal extubation; data are stored for further analysis as described before [17, 18]; and
- Manual ventilator settings and alarms, breath-by-breath alarm data, and any manual intervention at the ventilator is captured—this is done in the same way as described for the breath-by-breath ventilation data.

### Informed consent

All eligible patients will be informed about the study and asked for written informed consent before surgery. This will be done at the preoperative screening outpatient clinic, by telephone call or at admission the day before surgery. All approached patients will have sufficient time (at least 12 h) for consideration before the informed consent form is signed. No study-related actions will be performed before written informed consent is obtained. Informed consent will be documented on a paper form and signed by the patient and attending researcher. One signed informed consent form remains with the patient, the other will be stored in a designated secure folder in the respective participating center.

### Study dropouts and missing data

Subjects can leave the study at any time for any reason if they wish to do so without any consequences. The investigator can decide to withdraw a subject from the study for urgent medical reasons.

Subject will be classified as drop outs for the analyses of the endpoint “quality of ventilation” and “ICU team staff workload” if they did not receive postoperative ventilation for at least 2 h. This also means that if a patient is extubated in the operating room, that patient is considered a dropout. At no time a patient will remain intubated to reach at least 2 h of postoperative ventilation, i.e., extubation is never delayed for study purposes. A dropout rate of 5% has been accounted for in the sample size.

In case of withdrawal after randomization, the subject will not be replaced. Captured data until the moment of withdrawal will be stored and used for analysis. If the subject has not been randomized yet, the subject will be replaced to reach the intended sample size of patients.

### Handling of data

All of the patients directly identifying personal data (e.g., name, address, email, etc.) will be separated from the research data (e.g., measurement data, etc.) and replaced by an assigned code. The directly identifying data will be only used to contact the patients and only available to the local investigators. The handling of personal data complies with the general data protection regulations (GDPR) and applicable national regulations. To perform data collection and validation, an electronic case report form (eCRF), including validation checks and appropriate user access rights, will be set up in Castor EDC (www.castoredc.com). This application is a GCP compliant application that meets the standard for information security management. All data will be stored in a secure place for 15 years after termination of the study. Electronic files will be archived on a trial-designated folder on the cloud server of the respective participating center in a secure and controlled environment to maintain confidentiality. Electronic files will be controlled with password protection according to best practices. A GCP-certified monitor will monitor the study according to ICH–GCP guidelines throughout its duration. Any data leaks that might occur regardless of these safety precautions will be reported to all parties within one working day after discovery of the leak.

The objective of the clinical data management plan is to provide high-quality data by adopting standardized procedures to minimize the number of errors and missing data and, consequently, to generate an accurate database for analysis.

Accuracy and consistency checks will be carried out by way of automatic validation, pre-specified, and ad hoc checking by personnel at the coordinating centers. A qualified monitor will be installed to perform study monitoring according to the monitoring plan. Remote monitoring will be performed to signal early aberrant patterns, issues with consistency, credibility, and other anomalies. Onsite monitoring will comprise controlling presence and completeness of the research dossier and the informed consent forms, and source data checks will be performed as described in the monitoring plan.

Amendments are changes made to the research after a favorable opinion by the accredited Institutional Review Board (IRB) has been given. All amendments will be notified to the IRB that gave a favorable opinion. Non-substantial amendments will not be notified to the accredited IRB and the competent authority but will be recorded and filed by the sponsor. A “substantial amendment” is defined as an amendment to the terms of the METC application, or to the protocol or any other supporting documentation, that is likely to affect to a significant degree: the scientific value of the trial or the quality or safety of any intervention used in the trial. Any proposed amendments will first be notified to the sponsor. Following approval from the sponsor, the principal investigator (PI) will notify all participating centers of the changes. This ensures consistent implementation of the updated protocol across all trial sites. Any deviations from the protocol will be fully documented using a breach report form. This documentation will include the nature of the deviation, the reason for it, and any corrective actions taken. The protocol will be updated in the clinical trial registry to reflect any major changes.

### Sample size calculation

For the analysis of quality of ventilation, we need at least 110 patients in each arm of the study. We expect a dropout rate of 5%, as some patients may be liberated from the ventilator earlier. To correct for these dropouts, we propose to continue recruiting and randomizing patients until we have a sufficiently large sample size, i.e., we continue the study until we have 220 patients that have received postoperative ventilation for at least 2 h.

In regard of the secondary outcome, to demonstrate if automated ventilation by means of INTELLiVENT-ASV is non-inferior to non-automated ventilation by means of conventional ventilation with respect to duration of postoperative ventilation, we expect to need more patients for the analysis of this endpoint then for the other (primary) endpoints. Based on two recent randomized clinical trials in comparable patient groups, i.e., “POSITiVE” [17] and one comparing INTELLiVENT-ASV using mainstream end-tidal CO_2_ (etCO_2_) monitoring with INTELLiVENT-ASV using sidestream etCO_2_ monitoring (named “INTELLiSTREAM”) [18], we conservatively estimate to need 164 patients in each arm of the study (i.e., a total number of 328 patients is needed). The standard deviation for the duration of postoperative ventilation was found to be 3.9 h in POSITiVE [17] and 2.7 h in INTELLiSTREAM [18]. Based on these data, the true mean difference between INTELLiVENT-ASV and conventional ventilation in duration of postoperative ventilation is 1.5 h. Considering a standard deviation of 5.0 h (to account for imprecision), a true mean difference of − 1.01 (conservative estimate from POSITiVE, 70% of the effect in the study), an alpha of 0.025, a power of 90%, and equal allocation between the groups, therefore 164 patients in each arm is needed considering a margin of non-inferiority of ¾ h. Finally, a total of 328 patients will be included to the study.

### Statistical analysis

A full statistical analysis plan will be published online before the end of recruitment. All statistical analyses will be conducted according to the modified intention-to-treat principle considering all patients in the treatment groups to which they were randomly assigned, excluding cases lost to follow-up due to withdrawal of consent or cancelation of surgery. For both arms, the baseline characteristics will be expressed as counts and percentages, means and standard deviations, or medians and interquartile ranges, depending on normality of data distribution.

Data analysis will be performed blinded for the allocated study intervention. In all analyses, statistical uncertainties will be quantified with two-sided 95% confidence intervals. A two-sided *p*-value < 0.05 will be considered statistically significant. We will not adjust the *p*-value for multiple comparisons.

For the noninferioirty assessment of duration of ventilation, a one-sided noninferiority hypothesis test with a significance level of 0.025 and presented with a one-sided 97.5% confidence interval will be used. If noninferiority is confirmed, superiority will be tested considering a two-sided 95% confidence interval. Since the proposed approach will use a hierarchical closed-testing procedure examining a single confidence interval, no adjustment of the overall type I error will be done.

Statistical analysis will be performed using the free software program “R” (R Core Team, 2020, Vienna, Austria).

The effects of the intervention on quality of ventilation are analyzed and reported as done in previous studies of automated ventilation, i.e., the *time* spent in predefined zones of ventilation [17, 18]. As an add to the primary endpoint, next to *time* spent in predefined zones, we will also calculate *numbers* of breath in predefined zones. For details, see Appendix 1.

The impact of the intervention on ICU team staff workload is reported as in previous studies collecting data on alarms [12–14, 19].

All other endpoints, including duration of ventilation and ICU and hospital length of stay are reported as numbers and proportions.

Patient flows will be presented in a consolidated standard of reporting trials (CONSORT) flowchart.

Patients’ baseline characteristics will be presented per study arm. The proportion of patients who were treated according to their treatment assignment will be reported by treatment group.

Comparisons of the collected variables will be performed using *χ*^2^ tests for equal proportion, Student’s *t*-test for normally distributed data and Wilcoxon rank sum tests otherwise.

The database will be locked as soon as all data are entered and all discrepant or missing data are resolved—or if all efforts are employed and we consider that the remaining issues cannot be fixed. At this step, the data will be reviewed before database locking. After that, the study database will be locked and exported for statistical analysis. At this stage, permission for access to the database will be removed for all investigators, and the database will be archived.

No or minimally losses to follow-up for the primary and secondary outcomes are anticipated. Complete case analysis will be carried out for all the outcomes, that is, excluding patients with missing data in the outcome of interest. However, if any missing data is found for the primary outcome, a sensitivity analysis using multiple imputations and estimating equation methods will be performed.

### Subgroups analyses

There are no subgroup analyses planned, and a more detailed analysis plan will follow at the end of the study, before cleaning and closing of the database. The so-called “updated and finalized statistical analysis plan” will be outlined in a protocol amendment.

### Trial organization

The coordinating center and sponsor is the Medical University of Vienna. The steering committee is composed of the principal investigator, the coordinating investigator, the local investigators, and international experts of ventilation who contribute to the design and revisions of the study protocol.

The principal investigator, along with coordinating investigators, monitors, and auditors, will be granted access to the data and documents. A team of committed investigators will manage and coordinate patient recruitment and data collection daily. Investigators from the sponsor center will maintain frequent communication with other trial centers. Strict adherence to the trial protocol and relevant regulatory requirements will be ensured by the investigators.

An independent data safety monitoring board (DSMB), consisting of renowned, independent anesthesiologists, watches over the ethics of conducting the study in accordance with the Declaration of Helsinki and monitors safety parameters and the overall conduct of the study. The DSMB is composed of four independent experts.

The DSMB will meet after 50% of subjects are included or at least within 9 months after the first patient is enrolled. Subsequent to this meeting, the DSMB will meet virtually every 6 months. The DSMB will monitor safety by monitoring the specific safety endpoints as described above. The DSMB will monitor protocol compliance of both treatment strategies. The DSMB will review the overall status of the program: number of patients enrolled overall and, in each center, adherence to the protocol overall and by each center. All unexpected non-study related (S)AEs will be reported to the DSMB twice a year. Study-related SAEs will be sent to the DSMB, as soon as possible but at latest within 7 days after being received by the coordinating center. The advice of the DSMB will only be sent to the sponsor of the study. Should the sponsor decide not to fully implement the advice of the DSMB, the sponsor will send the advice to the reviewing medical ethical committee, including a note to substantiate why (part of) the advice of the DSMB will not be followed.

A safety analysis will be performed at 50% of patients included. Results of this safety analysis will be presented to the members of the DSMB and will be discussed at a planned meeting.

The study will be terminated if, as a result of our intervention, a disproportional amount of (serious) adverse events occur and causality between the intervention and adverse events is assumed. Interim analysis on safety will be performed and the results will be communicated, blinded for randomization, to the DSMB. Any deviation from the protocol, other than eight preapproved protocol deviations, are considered protocol violations. Protocol violations are to be reported and will be discussed with the DSMB.

## Discussion

POSITiVE II tests the hypothesis that automated postoperative ventilation by means of INTELLiVENT-ASV is superior to non-automated postoperative ventilation by means of pressure-controlled ventilation or pressure support ventilation with respect to quality of breathing *and* ICU team staff workload, and non-inferior with respect to duration of ventilation and lengths of stay in ICU in post-cardiac surgery patients. Thus far, there has been only one randomized clinical study that answered the question whether INTELLiVENT-ASV is superior to non-automated ventilation with respect to the quality of breathing [17]. This study, however, had a single-center design and preferably its findings are to be confirmed in an international, multicenter trial. We ourselves are performing another international multicenter trial, named ACTiVE, that will test a comparable hypothesis—a study that enrolls patients in the Netherlands and Switzerland [15]. ACTiVE is a study in general ICU population, and excluding patients included in this current study as one major inclusion criterium of ACTIVE is an expected need invasive ventilation for 24 h or more.

The automated mode tested in this study uses breath-by-breath adjustments for more lung-protective ventilation. With that, the risk for ventilator-induced lung injury could be reduced, eventually leading to better patient outcomes. We will capture breath-by-breath data to test the primary hypothesis.

In critically ill patients, even when ventilation last only some hours, it is recommended to use an appropriate low *V*_T_, to titrate PEEP and inspired fraction of oxygen by means of a PEEP/FiO_2_ table, to target a lower driving pressure, and to avoid both hyperoxia and hyperoxemia. This can be challenging and is time-consuming, especially when ICU teams are less experienced in invasive ventilation. Automated modes have the potential to reduce the time teams spent at the ventilator and the amount of alarms related to ventilation teams have to respond to––however, these modes may also be more difficult to set and “supervise”. One single-center randomized clinical study in Belgium [14] and single-center observational study in France showed that INTELLiVENT-ASV requires fewer manual interventions at the ventilator [13]. One recent single-center observational study in severely ill and difficult to ventilate COVID–19 patients in Switzerland showed that the number of manual interventions at the ventilator was half of that with conventional ventilation [12]. The second hypothesis tested, therefore, is that automated ventilation by means of INTELLiVENT-ASV reduces ICU team staff workload. For this, we capture data using a number of alarms and manual ventilator settings approach.

We will also compare clinically relevant clinical outcomes, duration of ventilation, and lengths of stay in the ICU. These endpoints are chosen for the following reasons: duration of ventilation can be seen as one important patient-centered endpoint: discomfort for patients is reduced with a shorter duration of ventilation—length of stay is an important ICU-centered endpoint, as an increase in ICU stay may translate in a lower number of ICU beds available for care for other patients, and higher costs. Of note, we do not expect a difference in duration of ventilation and lengths of stay in the ICU between the groups.

Ventilation in both groups is highly standardized, in the intraoperative phase during anesthesia and in the ICU when the patient is weaned from invasive ventilation. Other parts of care follow strict local clinical guidelines. POSITiVE II aims at minimizing bias by using concealed allocation and an intention-to-treat analysis with a pragmatic protocol that can be strictly adhered to. POSITiVE II is performed in several European countries, in different types of hospitals, making the results generalizable.

In summary, POSITIVE II is designed to confirm the findings of an earlier single-center study, whether automated ventilation by means of INTELLiVENT-ASV is superior to non-automated ventilation by means of conventional ventilation, with respect to quality of breathing and ICU team staff workload. The results of this study can help ICU teams in their choices regarding the use of automated ventilation in the postoperative care setting.

## Trial status

Protocol version 1.2; January 29, 2024.

Recruitment: Estimated recruitment start will be on May 1, 2024. The anticipated recruitment completion is in August 2025.

### Supplementary Information

Supplementary Material 1. Spirit checklist.

Supplementary Material 2. Appendices.

## Data Availability

The full trial protocol and informed consent materials are available on any reasonable request to the corresponding author. The final trial dataset and the code for statistical analysis will be accessible by non-commercial partners upon request. A full monitoring plan is obtainable on reasonable request.

## Abbreviations

AE: Adverse event
ARDS: Acute respiratory distress syndrome
CO_2_: Carbon dioxide
COPD: Chronic obstructive pulmonary disease
(e)CRF: (Electronic) case record form
DSMB: Data safety monitoring board
ERAS: Enhanced recovery after surgery
etCO_2_: End-tidal carbon dioxide
FeV1: Forced expiratory volume in the first second
FiO_2_: Fraction of inspired oxygen
GCP: Good Clinical Practice
GDPR: General Data Protection Regulation
ICU: Intensive care unit
INTELLiVENT-ASV: INTELLiVENT-adaptive support ventilation
“INTELLiSTREAM”: INTELLiVENT-ASV using mainstream versus sidestream capnography in cardiac surgery patients’ study
H_2_O: Water
ΔP: Driving pressure
PaCO_2_: Partial pressure of carbon dioxide in the arterial blood
PaO_2_: Partial pressure of oxygen in the arterial blood
PBW: Predicted body weight
PEEP: Positive end-expiratory pressure
*P*max: Maximum airway pressure
POSITiVE: POStoperative INTELLiVENT-adaptive support VEntilation in cardiac surgery patients’ study
POSITiVE II: POStoperative INTELLiVENT-adaptive support VEntilation in cardiac surgery patients II study
RR: Respiratory rate
SAE: Serious adverse event
SaO_2_: Arterial oxygen saturation
SpO_2_: Pulse oximetry oxygen saturation
TOF: Train-of-four
VC: Vital capacity
*V*_T_: Tidal volume
