# Supplementary material for: POStoperative INTELLiVENT-adaptive support VEntilation in cardiac surgery patients (POSITiVE) II—study protocol of a randomized clinical trial
Source: Trials. 2024 Jul 3;25:449. doi: 10.1186/s13063-024-08296-2 (PMC11223327; doi:10.1186/s13063-024-08296-2)
Supplement: Supplementary file 2 — Supplementary Material 2. Appendices. [file 13063_2024_8296_MOESM2_ESM.docx]

## Appendix 1. Zones of ventilation (used for primary endpoint, quality of ventilation)

The primary outcome is the proportion of time spent in three predefined and previously used zones of ventilation in the first 2 hours of postoperative ventilation, according to 4 ventilation parameters, including tidal volume (V_T_), maximum airway pressure (Pmax), end–tidal carbon dioxide (etCO_2_) and oxygen saturation by pulse oximetry (SpO_2_) (**Table 1**). One secondary endpoint is the proportion of breath spent in these zones of ventilation. Likewise, we will define zones according to other sets of ventilation parameters, for lung protection (V_T_, PEEP, respiratory rate (RR), and Pmax), for gas oxygenation (SpO_2_, PEEP, and FiO_2_), for decarboxylation (etCO_2_, minute ventilation, and deadspace), and for energytrauma (driving pressure, and mechanical power).

| **Table 1** The predefined ventilation zones, adapted from de Bie et al (15) | | | | |
| --- | --- | --- | --- | --- |
| parameters | | critical | acceptable | optimal |
| V_T_, ml/kg PBW | | > 12 | 8-12 | ≤ 8 |
|  | | *OR* | *OR* | *AND* |
| Pmax, cm H_2_O | | ≥ 36 | 31 – 36 | ≤ 30 |
|  | | *OR* | *OR* | *AND* |
| etCO_2_, mmHg | | < 25 OR ≥ 51 | 25 – 30 OR 46 – 51 | 30 – 46 |
|  | | *OR* | *OR* | *AND* |
| SpO_2_, % | | < 85 | ≥ 98 *OR* 85 – 93 | 93 – 98 *OR* ≥ 93 if FiO_2_ ≤ 40% |
| Definitions | A breath is considered ‘critical: if any parameters are in ‘critical zone’; a breath is considered ‘acceptable’: no parameters are in ‘critical zone’, but not all parameters in ‘optimal zone’; a breath is considered ‘optimal’: if all parameters in ‘optimal zone’; if a parameter is missing, a breath is defined as ‘missing’. | | | |
| Abbreviations: V_T_, tidal volume, PBW, predicted body weight; Pmax, maximum airway pressure; et–CO_2_: end–tidal carbon dioxide; FiO_2_: fraction of inspired oxygen; SpO_2_: oxygen saturation by pulse oximetry. | | | | |

## Appendix 2. Workload observations

A main secondary endpoint is ICU nursing staff workload, which is captured by the ventilator software collecting data on alarms during postoperative care in the ICU

- Number of alarms
- Types of alarm
- Duration of alarm
- Responses to alarm
- Alarm settings and adjustments
- Breath–by–breath alarm data
- Any manual intervention at the ventilator
